# Bipolar Disorder and Manic-Like Symptoms in Alzheimer’s, Vascular and Frontotemporal Dementia: A Systematic Review

**DOI:** 10.2174/1570159X20666220706110157

**Published:** 2023-09-25

**Authors:** Camilla Elefante, Giulio Emilio Brancati, Samuele Torrigiani, Salvatore Amadori, Sara Ricciardulli, Gabriele Pistolesi, Lorenzo Lattanzi, Giulio Perugi

**Affiliations:** 1 Department of Clinical and Experimental Medicine, University of Pisa, Psychiatry Unit, Pisa, Italy;; 2 Psychiatry Unit, Azienda Ospedaliero-Universitaria Pisana, Pisa, Italy;; 3 G. De Lisio Institute of Behavioral Sciences, Pisa, Italy

**Keywords:** Mania, bipolar disorder, manic-like symptoms, dementia, mild cognitive impairment, Alzheimer, vascular dementia, frontotemporal dementia

## Abstract

**Background:**

An increased risk of manic episodes has been reported in patients with neurodegenerative disorders, but the clinical features of bipolar disorder (BD) in different subtypes of dementia have not been thoroughly investigated.

**Objectives:**

The main aim of this study is to systematically review clinical and therapeutic evidence about manic syndromes in patients with Alzheimer’s disease (AD), vascular dementia (VaD), and frontotemporal dementia (FTD). Since manic-mixed episodes have been associated to negative outcomes in patients with dementia and often require medical intervention, we also critically summarized selected studies with relevance for the treatment of mania in patients with cognitive decline.

**Methods:**

A systematic review of the literature was conducted according to PRISMA guidelines. PubMed, Scopus, and Web of Science databases were searched up to February 2022. Sixty-one articles on patients with AD, VaD, or FTD and BD or (hypo) mania have been included.

**Results:**

Manic symptoms seem to be associated to disease progression in AD, have a greatly variable temporal relationship with cognitive decline in VaD, and frequently coincide with or precede cognitive impairment in FTD. Overall, mood stabilizers, and electroconvulsive therapy may be the most effective treatments, while the benefits of short-term treatment with antipsychotic agents must be balanced with the associated risks. Importantly, low-dose lithium salts may exert neuroprotective activity in patients with AD.

**Conclusion:**

Prevalence, course, and characteristics of manic syndromes in patients with dementia may be differentially affected by the nature of the underlying neurodegenerative conditions.

## 1 INTRODUCTION

A bidirectional link between bipolar disorder (BD) and dementia has been suggested [1-3], however, epidemiology, pathogenic hypotheses, clinical presentations, and pharmacological treatments of BD in patients with different subtypes of dementia have been scarcely explored. According to a recent meta-analysis, a lifetime diagnosis of BD confers a greater vulnerability of dementia compared to a history of depression [2]. On the other hand, a cohort study based on Danish hospital registers showed an increased risk of developing mania (or first bipolar episode) in patients with dementia compared to other chronic and disabling medical illnesses, suggesting a common neuropathological substrate shared between the two disorders [4]. Ng *et al*. [1] have tried to explain the relationship between dementia and BD assuming that the neurodegenerative process could unveil bipolarity in patients with a predisposing diathesis. In this perspective, mixed-labile manic and depressive states and atypical depressions emerging in the setting of dementia might be interpreted as a less penetrant form of bipolarity. This putative late-onset bipolar spectrum disorder called by the Authors BD type VI was supported by a case series of patients with sub-bipolar temperaments and/or a predisposing familial diathesis developing mood dysregulation after the onset of a neurodegenerative process [1]. The prevalence of BD seems to be different depending on the dementia subtype considered. In a large healthcare system population study, patients with frontotemporal dementia (FTD) showed the highest 2-year prevalence of BD (5.67%), while patients with Alzheimer’s disease (AD) showed the lowest (1.33%) and patients with vascular dementia (VaD) had an intermediate rate (3.36%) [3]. However, while in clinical studies patients are mostly recruited in the initial phases of dementia, manic symptoms, as well as other psychiatric symptoms, could be evident at different stages in the course of different diseases [5], possibly leading to an underestimation of BD in some dementia subtypes. The aim of this review is to critically summarize the evidence on clinical features of BD and manic-mixed episodes occurring in the setting of different dementia subtypes, specifically in patients with AD, VaD, and FTD. Since for AD there was little evidence about mania, we have also examined studies about manic-like neuropsychiatric symptoms. Possible therapeutic implications of BD in these patients are also discussed.

## 2 MATERIALS AND METHODS

### 2.1 Search

A systematic review of the literature was conducted and the Preferred Reporting Items for Systematic Reviews and Meta-Analyses (PRISMA) guidelines were used to describe procedures and results [6]. PubMed, Scopus, and Web of Science bibliographic databases were searched from their date of inception to February 6^th^, 2022. The research team discussed and reviewed the results of an initial scoping search. We developed a strategy using four groups of search terms. These were: ‘dementia’ or ‘cognitive impairment’ or ‘MCI’ or ‘cognitive decline’ or ‘Alzheimer’ or ‘FTD’ or ‘Pick’ (group 1) AND ‘mania’ or ‘manic’ or ‘hypomania’ or ‘hypomanic’ or ‘bipolar disorder’ or ‘mixed depression’ or ‘bipolarity’ (group 2). Additional terms, namely ‘Huntington’, ‘Lewy bodies’, ‘Parkinson’, ‘corticobasal degeneration’, ‘progressive supranuclear palsy’, ‘pseudodementia’ or ‘reversible dementia’ were included in group 1 to broaden the search to retrieve studies focusing on other neurodegenerative disorders but possibly including patients with AD, VaD or FTD. Terms were adapted as necessary for each database. Results were downloaded into Mendeley software. The search included reviews and original studies. If a previous review was found, we searched the reference list to identify and retrieve the primary studies. Reference lists of included studies were also carefully searched for relevant citations.

### 2.2 Eligibility Criteria

Only original studies were eligible for our review. No restriction for study design or group comparisons was applied. In order to be included in our review, study participants or subgroups of participants should have been diagnosed with AD, VaD or mixed dementia, FTD and BD, mania, hypomania, or mixed depression. There were no age or age-at-onset limits for either dementia or BD. Studies on neurobiological correlates and underpinnings of dementia and/or BD were excluded. All studies on clinical features, course, and treatment of patients who were cross-sectionally or longitudinally diagnosed with BD and AD, VaD or mixed dementia, or FTD were included. Due to the paucity of data on mania in AD, we selected all the retrospective, cross-sectional, and longitudinal studies with more than 50 patients, in which manic or hypomanic symptoms were explored by means of the Neuropsychiatric Inventory (NPI), both adopting the NPI-10 [7] and the NPI-12 [8] versions.

### 2.3 Abstract Screening and Study Selection

**Fig. (1):**
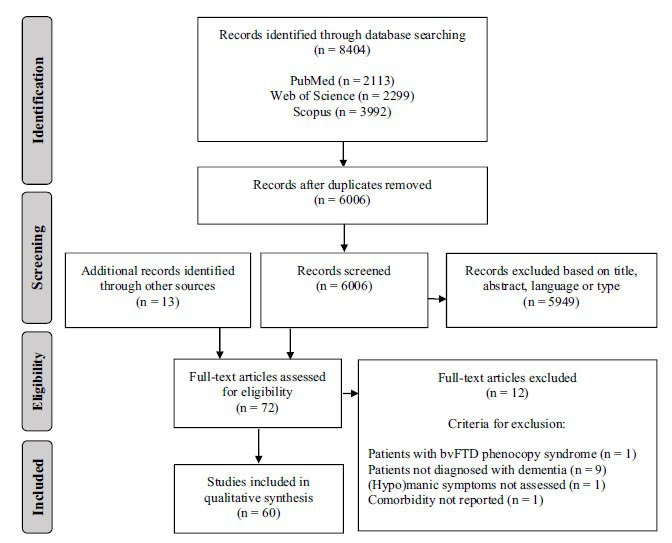
PRISMA flowchart showing the process of identification and selection of studies.

8404 abstracts were retrieved using our search strategy, of which 2398 were removed as duplicates. Thus, 6006 abstracts were screened. If a title appeared potentially eligible, but no abstract was available, the full-text article was retrieved. Two researchers (CE and GEB) scanned all titles and abstracts to identify relevant articles for full-text retrieval. Disagreements were resolved by discussion after consulting a third reviewer (GP). 5949 records were excluded based solely on the title or abstract and 59 full-text articles were retrieved. 13 additional records were identified through other sources (citations in reference lists of screened papers and reviews) and assessed for eligibility. A total of 72 full-text articles were thoroughly assessed for eligibility. 60 studies were finally included in the systematic review and 12 articles were excluded (Fig. **1**).

## 3 BIPOLAR DISORDER AND MANIC-LIKE SYMPTOMS IN PATIENTS WITH DEMENTIA

Overall, 60 studies were included in our systematic review on BD in patients with dementia and discussed hereafter, including 15 studies on patients with AD, 5 of which focused on drug-induced mania, 12 on patients with VaD, 28 on patients with FTD, and 5 additional studies on lithium treatment in patients with AD or FTD. Since very few studies focused on treatments for BD in patients with dementia and since a systematic review of treatments for dementia-related behavioral disorders and psychosis was beyond the scope of this article, we narratively reviewed selected studies of interest for pharmacological treatment of BD in patients with dementia in a separate section.

### 3.1 Manic Syndromes and Manic-like Neuropsychiatric Symptoms in Alzheimer’s Disease

AD is the most prevalent cause of dementia accounting, alone or in combination with other disorders, for approximately 60-80% of all cases [9]. Depression is very frequent from the early stages of AD having a prevalence of almost 50% in mild AD [10, 11]. Mania, instead, has been estimated to be about 3% in hospitalized patients [12], while less than half of that prevalence has been reported in mixed samples of outpatients and inpatients [3]. Differently from what happens in FTD, in which new-onset mania frequently occurs first [13, 14], it is possible that mania tends to occur at the final stages of dementia in AD and so its prevalence could have been underestimated so far. In fact, end-stage AD patients are often institutionalized and therefore made unavailable to take part in clinical studies. Another possible explanation for the low frequency of mania may be due to the localization of neuropathological alterations in AD, that relatively preserve areas typically involved in the genesis of manic symptoms, such as the frontal lobe [15].

Since only a few studies investigated the occurrence of (hypo)manic episodes in patients with AD [3, 12], exclusively for this type of dementia, we decided to examine the prevalence and timing of the appearance of manic-like neuropsychiatric symptoms (NPSs) assessed by means of NPI. For this purpose, we considered eight studies: three studies [16-18] evaluating manic-like behaviors through NPI met the criteria for full-text review and five articles were further included by hand-searching from reference lists of potentially eligible studies (Table **1**) [19-23]. In all the studies, the authors tried to group individual NPSs reclassifying them into distinct psychiatric syndromes. The reduction of the 12 or 10 original NPI-items into a lower number of dimensions by means of factor-analysis led to greater efficacy in finding correlations among behavioral disturbances and clinical variables [19]. Euphoria and disinhibition contributed to defining the manic syndrome in seven out of eight studies, the large majority of which (five of seven) have considered euphoria and disinhibition alone as identifying manic, hypomanic and “frontal” syndromes [16, 18, 19, 21, 24]. In the remaining two studies euphoria and disinhibition have been found to frequently occur together with other symptoms. Specifically, in the study by Hollingworth *et al*. [23], euphoria and disinhibition have been included with aberrant motor behavior and sleep/appetite disturbances into a “behavioral dyscontrol” factor, whereas in the study by Aalten *et al*. [22] they were associated to aberrant motor behavior, agitation, and irritability to form a “hyperactivity” factor. Euphoria and disinhibition, instead, did not cluster within the same symptom factor in the study conducted by Fuh *et al*. [20]. Possibly due to the inadequate sample size used in this last study, the euphoria has been included in a factor called “psychomotor regulation” together with agitation and irritability, whereas disinhibition was loaded on a “social engagement” factor together with apathy. As for other less specific manic symptoms, agitation, and irritability were considered in most studies manifestations of excitement, occurring together with euphoria and/or disinhibition [20, 22] or with other psychomotor disturbances [17, 18, 23]. However, in other studies, agitation and irritability have been interpreted as mostly related to other symptoms, such as depression [16], mood disturbances [21], psychosis [19], and hallucinations [21], suggesting a potential link among these features in AD patients, as already observed by other authors [25, 26].

When looking at the prevalence of euphoria and disinhibition, the symptoms corresponding to the manic syndrome in most of the studies, we could observe that higher rates were found in groups with lower MMSE scores, suggesting that the prevalence of manic-like symptoms increases with the severity of dementia. Within samples with low mean MMSE score, *i.e.* between 12.7 and 13.3 (moderately/severe AD), the prevalence of manic-like neuropsychiatric symptoms ranged from 5% to 26% for euphoria and from 20.4% to 34% for disinhibition [16, 19, 20, 23]. Lower rates (4.5-7% for euphoria, 10.2-12.6% for disinhibition, and 7.7-7.9% for euphoria and disinhibition) were found in samples with higher mean MMSE scores, *i.e.* between 18.2 and 23.4 (mild and moderate AD) [17, 18, 21, 22]. The sample with an intermediate mean MMSE score (16.7) had an intermediate prevalence of manic-like symptoms (9.8% for euphoria, 13.3% for disinhibition) compared to the other two groups [21]. Several authors, independently from each other, reported the association between higher rates of manic-like neuropsychiatric symptoms and increased severity of cognitive impairment [18, 20, 21]. In two samples, not only the prevalence but also the severity of manic-like symptoms was positively associated to cognitive decline [18, 23].

An observational longitudinal study found a greater worsening of cognition in patients with manic-like symptoms compared to those without [17]. On the contrary, one study showed a slower rate of progression (MMSE points/year) in patients with manic-like symptoms compared to the rest of the sample. However, in this latter study, the subsample with manic-like symptoms had a higher level of education, which may have relatively mitigated the rapidity of cognitive decline [19].

### 3.2 Mania Induced by Acetylcholinesterase Inhibitors

Six cases of manic episodes associated to the use of acetylcholinesterase inhibitors (AChEIs) in patients with probable AD have been reported (Table **2**) [27-31], two of which also had vascular alterations [29, 30]. Three of these subjects had been previously diagnosed with BD type I [27, 29], one of which had a late-onset [29], and two subjects had previous depressive symptoms [30, 31], in one case arising during treatment with AChEIs, just before the occurrence of mania [31]. Only one patient had no personal history of psychiatric disorders [28]. Type and the daily dose of AChEIs varied, including rivastigmine 3 to 4.6 mg/day, galantamine 8 to 16 mg/day, and donepezil 10 mg/day. In five out of six cases manic symptoms started from 3 days to 2 weeks after the initiation/optimization of AChEI treatment, supporting the existence of a relationship between the onset of mania and treatment [27-30]. The latter patient developed mood elevation many months after donepezil introduction [31]. At the onset of mania, three patients were simultaneously taking an antidepressant therapy [27, 30]. Despite antidepressants are generally known to increase the risk for mania, each of these subjects had received the antidepressant drug for a relatively long period (1 to 8 years) without showing the emergence of clinically relevant manic symptoms, supporting the possibility of an AChEI-induced mania. In all cases AChEIs and, whenever present, antidepressants were discontinued [27-31]. The remission of mania was observed after a period ranging from 2 days to 4 weeks, either spontaneously [27, 28, 31] or following treatment with antipsychotic and/or antiepileptics [29, 30]. In one patient, who initially developed mania on rivastigmine, no additional manic symptoms were reported after re-challenging with donepezil 2.5 mg/day, possibly as a consequence of the lower dose administered [28].

Although the number of observed AD patients developing mania with AChEI treatment is small and the pathophysiological mechanism is not clear, the close temporal relationship between onset and improvement of manic episodes with administration and discontinuation of AChEIs suggests the existence of a causal link. Moreover, the effect of AChEIs on mood is not limited to patients with AD, but it has been reported in patients affected by different brain neurological illnesses, from neurocognitive disorders to cerebrovascular diseases [32-37]. It is plausible that previous mood disorders or subclinical mood symptoms associated to neuropathological alterations confer a vulnerability to mood elevation with AChEI use. This represents a class effect, since each type of AChEI could be associated to the emergence of manic symptoms in patients with neurodegenerative diseases, although low doses may be more tolerable. The evaluation of the risk of mania and concomitant treatments is essential before the introduction of AChEIs therapy in people with AD and a bipolar diathesis.

### 3.3 Manic Syndromes in Vascular Dementia

VaD is the most common cause of dementia after AD, accounting for 15% of all cases [38]. VaD is not a clearly validated diagnostic entity, but this term refers rather to a heterogeneous group of disorders. The most common forms of VaD in the elderly are multi-infarct dementia, strategic infarct dementia, and subcortical VaD [39]. Multi-infarct dementia and strategic infarct dementia relate to large vessel disease, whereas subcortical VaD, which incorporates the old entity “Binswanger’s disease” [40], is the consequence of lacunar infarcts, focal and diffuse ischemic white matter lesions, and incomplete ischemic injury related to small vessel disease [41]. Lesions similar to those of subcortical VaD can also be found in cerebral autosomal dominant arteriopathy with subcortical infarcts and leukoencephalopathy (CADASIL) [42]. The term vascular cognitive impairment (VCI) has been proposed to refer to the full range of cognitive deficits from mild cognitive impairment (MCI) to dementia associated to cerebrovascular disease [43-45].

Cerebrovascular alterations have also been correlated with increased vulnerability to a variety of psychiatric syndromes and mood disorders. Alexopoulos *et al*. [46] first formulated the vascular depression hypothesis, according to which “cerebrovascular disease may predispose, precipitate, or perpetuate a depressive syndrome in older adults”. The presence of subcortical vascular lesions in geriatric depressed patients have been associated to motor retardation, apathy, scarce depressive ideation, executive dysfunctions, unstable remission, and poor response to antidepressants [47].

On the other side, the term vascular mania has been mostly employed to refer to patients developing manic symptoms within a month after a stroke [48]. However, a causal relationship between stroke and mania has been hypothesized also for patients developing mania many months or even some years after a cerebrovascular event [49]. In the present review, we have collected cases of patients that develop both cognitive impairment and mania following a cerebrovascular disease regardless of how much time has elapsed between the ischemic event, the occurrence of cognitive symptoms, and the onset of mania. We suppose, in fact, that mania due to a cerebrovascular disease does not necessarily become evident in the short term but may have latency and emerge as a result of further clinical or sub-clinical ischemic events and/or after the occurrence of other predisposing factors such as the aging process. After excluding a case report in which vascular damage has not been ascertained [50], seven cases met the criteria for full-text review [37, 51-55], and five more were included by hand-searching from reference lists [56-60]. Patients have been grouped into cortico-subcortical VCI due to large vessel disease, subcortical VCI due to small vessel disease, and VCI due to CADASIL disease (Table **3**).

Except for two patients who had the first manic episode during their 40s [55, 57], all the other patients were aged ≥ 50 years at the time of the first manic or hypomanic episode. Despite a significant relationship between post-stroke mania and right hemispheric lesions have been established [48], in our sample two out of four patients with cortical infarcts had left-sided lesions [52, 57]. This result supports the fact that post-stroke mania is not exclusively associated to lesions in the right hemisphere, as already indicated by other reports of mania following left lesions documented in the literature [61-63].

The temporal relationship between the development of VCI and the onset of mania was highly variable among the subgroups. In the cortico-subcortical subgroup, two patients with multiple infarcts had a manic onset in the same period of the first cognitive alterations [37, 55] and two patients, who had infarcts respectively in the frontal and frontotemporal cortex due to large vessel disease, showed manic symptoms some months after the development of overt dementia [52, 57]. Differently, in all patients having dementia due to small vessels, including those with CADASIL, manic symptoms occurred clearly before the diagnosis of dementia, when the subjects had little or no evidence of cognitive impairment. This tendency is marked in patients with CADASIL, the majority of whom showed only some cognitive deficits at the onset of mania, mainly in the executive domain. According to this trend, we could suppose that, given the role of frontostriatal circuits in mood regulation [64, 65], fewer vascular alterations in subcortical brain regions due to small vessel disease are sufficient to induce mood instability, as also supported by studies on late-life depression [47], while a greater number of lesions is needed to produce significant cognitive dysfunctions at later stages. The fact that late-onset mania may occur, before the development of dementia, in subjects with mild cognitive alterations or normal cognition underlines the need to routinely acquire brain images in order to exclude brain damage in all patients with an onset of mania outside the usual age range, as already indicated by some authors [66].

When looking at psychiatric antecedents, none of the patients belonging to the cortico-subcortical VCI group showed a positive family history of psychiatric disorders, whereas one out of two patients belonging to the subcortical VCI group [56] and one out of six patients belonging to the CADASIL subgroup had a positive psychiatric family history, specifically of depression [53]. In summary, most patients had no positive psychiatric family history and none of them had a family history of BD. Conversely, all patients belonging to the cortico-subcortical VCI subgroup had a personal history of other mood episodes before mania [37, 52, 55, 57]. Two patients have already had recurrent depressive episodes early in life [37, 57], one has had a depressive episode at the age of 40 and after 2 years developed hypomania, and another patient has already had a hypomanic episode at the age of 50 [52]. In the cortico-subcortical subgroup, it seems that the emergence of VCI could confer vulnerability to BD in patients with previous depression [37, 55, 57] and could trigger the transition from hypomania to mania as observed in a patient with BD type II [52]. Differently, none of the patients with subcortical VCI had early-onset mood disorder before vascular mania and one of them had a late-onset depression shortly before the onset of mania [56]. Psychiatric history before mania was not homogeneous for the subgroup with CADASIL: some patients had no previous psychiatric disorders [54, 59], other patients previously showed personality changes [53, 60] and alcohol abuse [58, 60], and one patient had a history of depression [60]. Overall, most patients were taking antidepressants or stimulants at the time of manic onset [37, 53, 55, 60, 67]. However, manic symptoms cannot be considered merely drug-induced since these treatments had been introduced long before the manic episode in all these cases.

From a clinical point of view, it seems noteworthy that mixed features like psychomotor retardation and mood lability were present in four out of twelve patients [51, 53, 57, 68] and psychotic symptoms like persecutory and jealousy delusions, auditory/visual hallucinations in three out of twelve patients [56, 57, 60], one of which also showed mixed symptoms [57], confirming Ng *et al*. [1] suggestion that mixed features are the typical presentation of mania in the context of dementia.

Finally, information on treatment was available for nine patients. Treatment was based, as previously reported [48], on the introduction of mood stabilizers and/or antipsychotics and the discontinuation of eventual antidepressants and/or stimulants. In five patients mood stabilizers, alone or in combination with antipsychotics, have been effective in the treatment of mania [37, 54, 57, 60, 68]. Monotherapy with antipsychotics has shown limited efficacy. In fact, in two patients that have been unsuccessfully treated with antipsychotics, the resolution of mania followed the introduction of mood stabilizers [37, 54]. Just one patient, who has had only a partial response to mood stabilizers, showed a full response to antipsychotics [56]. Therapies for cognition, specifically donepezil and memantine have been administered in two patients, one of which showed mood instability with both donepezil and memantine [37]. Another patient did not show any mood alterations after administration of donepezil [57].

### 3.4 Bipolar Disorder and Frontotemporal Dementia

FTD is the second most common dementia in the presenile population after AD. Three main subtypes have been described, namely the behavioral variant (bvFTD), previously known as Pick's disease, and two variants of primary progressive aphasia known as semantic (svPPA) and non-fluent variants (nfvPPA). Psychiatric misdiagnoses, especially with BD, occur in up to 50% of bvFTD patients and, on average, diagnosis is delayed up to 5-6 years after the onset of symptomatology [69]. On the other hand, bvFTD can also be misdiagnosed in patients with psychiatric disorders. Indeed, up to one-third of patients initially diagnosed with possible or probable bvFTD can be reclassified as a psychiatric disorder after two years of multidisciplinary follow-up [70]. However, other authors observed that formally diagnosed psychiatric disorders are not overrepresented in patients with probable bvFTD, suggesting that psychiatric misdiagnosis in bvFTD can be reduced by strictly applying diagnostic criteria [71]. From a clinical point of view, the differential diagnosis between FTD and psychiatric disorders may be based on longitudinal trajectories of frontal and stereotyped behaviors, general and frontal cognitive functioning, and social cognition: while frontal behavioral symptoms, such as disinhibition or apathy, worsen over time in patients with bvFTD, usually improve in patients with psychiatric disorders or remain stable in other neurodegenerative disorders [72].

FTD symptoms frequently overlap with those of BD, including euphoria and elation [73], irritability, increased energy, pressured speech, racing thoughts, task-oriented behavior, psychomotor hyperactivity, as well as, distractibility and disinhibition [74]. Shared origins and mechanisms between these conditions have been previously hypothesized and approximately 10% of patients with bvFTD also meet the criteria for BD [13]. Manic or hypomanic episodes may precede other symptoms of FTD by many years, possibly representing either a risk factor for FTD or a prodrome associated to early anterior temporal involvement. Seven cases of patients who were diagnosed with BD in young adulthood (< 40 years) and subsequently developed FTD have been identified through our search (Table **4**) [75-80]. In three cases the onset of dementia was suggested by the occurrence of neurological signs, including poor coordination and language impairment [75], or progressive neuropsychiatric alterations (impulsivity, irritability, short-term memory loss, perambulation, persecutory delusions, disorientation) [78]. Another patient presented with behavioral symptoms suggesting FTD, such as less attention to hygiene and personal care, poor inhibition, and decreased the ability to regulate social behavior [76]. Intriguingly, in one patient, the onset of FTD dated back to a manic episode which was followed by progressive cognitive impairments [77].

On the other hand, BD could represent the first manifestation of FTD, especially when occurring late in life. Five cases of late-onset BD (> 40 years) that, after a period of time ranging from 10 to 20 years, were diagnosed with bvFTD, were previously reported (Table **4**) [80-84]. Neurological signs [80] or apathy, social withdrawal and hyperphagia [83] gave rise to suspect FTD in two cases. The other three patients have been investigated for dementia following the development of a manic episode with atypical features such as hypersexual talk, public masturbation, and delusional fixed ideas with repetitive behaviors, which were not present in previous affective episodes [81, 82, 84]. In ten additional cases, FTD onset coincided with a first manic episode which satisfied usual diagnostic criteria (*e.g.* euphoria, racing thoughts, logorrhea, increased energy, reduced need for sleep, prodigality, and grandiosity), but also showed atypical symptoms such as disorganization and disinhibited or childish behaviors [85-94]. One of these patients had presented two years earlier with an episode characterized by apathy, anhedonia, lack of energy, and empathy [87], while the other six patients had experienced similar symptomatology about a year after the manic episode [85, 86, 88, 89, 92, 93].

Interestingly, three patients out of fifteen presenting with mania before FTD diagnosis had C9orf72 mutated [84, 88, 92]. The other two patients had a mutation in the GRN gene [80, 83]. Finally, one patient presented a polymorphism associated to FTD on exon 12 of chromosome 17 [75]. At least in a subgroup of patients, FTD possibly shares a genetic predisposition with BD. In particular, the expansion of C9orf72 has been reported in a patient diagnosed with BD, one of whose parent with the same mutation showed an atypical and late-onset BD and subsequently progressed to FTD [95]. Another BD patient, who showed the same expansion and whose father was diagnosed with AD, has been described [96]. Even though BD was not found to be more prevalent in relatives of probands with FTD or amyotrophic lateral sclerosis who carried the C9orf72 mutation, higher rates of psychotic and mood disorders, suicide, and autism spectrum disorders were observed in C9orf72 carriers compared to non-carriers [97]. Further studies specifically assessing the prevalence of BD in samples of FTD patients and relatives showing different underlying mutations are warranted.

Further complicating the relationship between FTD and BD, bipolar patients may also show progressive cognitive and behavioral impairments that mimic bvFTD, constituting part of non-progressive FTD phenocopy syndromes [98]. Four cases of BD patients who gradually developed a syndrome fulfilling the criteria for possible bvFTD, including apathy, disinhibition, loss of empathy, stereotypical behavior, and compulsiveness, in which 3- to 7- years follow-ups yielded no clinical progression, have been described by Dols and colleagues [99]. Repeated neuroimaging was within normal limits, cerebrospinal fluid biomarker studies were not supportive of underlying neurodegenerative pathology and C9orf72 mutation status was negative in all cases [99]. Some authors also hypothesized a specific post-bipolar dementia syndrome similar to bvFTD in several aspects, but only associated to mild longitudinal worsening [100].

As for the treatments available, lithium salts have not been evaluated in placebo-controlled trials to treat agitation/aggression with or without psychosis in FTD. However, one case series found low-dose lithium carbonate to significantly improve agitation and aggression in three patients with FTD [101]. Antipsychotics are often used to control agitation, aggression, and disinhibition in patients with FTD, despite this population is highly sensitive to the extrapyramidal side effects [102]. Serotoninergic drugs on their part showed some efficacy on behavioral symptoms of FTD. Treatment with citalopram 40 mg/day has been found to be effective on disinhibition, irritability, and depressive symptoms of FTD [103], but a systematic review on pharmacological interventions for bvFTD concluded that trazodone has the greatest effect on behavioural disturbances including depressive symptoms, insomnia, irritability, and agitation [104]. Compared to the use of atypical antipsychotics, the use of trazodone is associated to a lower rate of mortality in patients with dementia, though trazodone is not completely safe in this population. In fact, the risk of fractures and falls is similar in patients treated with trazodone or atypical antipsychotics [105]. Besides, AChEIs are not recommended for the treatments of behavioral symptoms in FTD [104]. AChEIs use, in fact, may exacerbate behavioral symptoms [106]. One study reported that the donepezil discontinuation led to improvement in agitation/aggression, irritability/lability, aberrant behavior, and delusions [107]. Finally, preliminary studies with stimulant drugs have been conducted on the basis of the hypothesis of dopaminergic dysfunction in FTD [108]. Dextroamphetamine has shown potential efficacy in reducing apathy and disinhibition in eight patients with bvFTD [109] and a single administration of methylphenidate (40 mg) effectively reduced abnormal risk-taking behavior in a small sample of bvFTD patients [110]. However, mania has been triggered by levodopa in a patient with a C9orf72 mutation associated bvFTD [111], suggesting that caution is needed in the introduction of dopaminergic treatments in subjects with FTD.

## 4 PHARMACOLOGICAL AND PHYSICAL TREATMENT OF (HYPO)MANIC SYMPTOMS IN PATIENTS WITH DEMENTIA

No randomized controlled trials of treatments for BD in patients with dementia have been conducted so far. Consequently, the evidence for the efficacy and tolerability of pharmacological treatments for mania in patients with dementia is mainly based on case reports and trials on behavioral symptoms of dementia. An improvement in behavioral symptoms of dementia, including agitation and aggression, has been observed in patients with mild to moderate AD after treatment with specific drugs, especially memantine, and rivastigmine [112]. However, no specific treatments for dementia-related manic symptoms have been proposed. Indeed, patients with dementia and behavioral disorders, such as agitation or aggression, and psychotic symptoms are likely to receive antipsychotics, notwithstanding the increased risk of adverse outcomes. However, whenever possible, non-pharmacologic interventions and mood stabilizers should be preferred. Anticonvulsants, such as valproate, have been widely used in clinical practice for patients with manic symptoms and lithium salts still represent a good option, despite the possibility of neurotoxicity at serum levels within the therapeutic range due to a combination of neuropathological alterations, medical comorbidities, and age‐related pharmacokinetic changes in patients with dementia.

### 4.1 Lithium

Lithium is the pharmacological agent with the oldest use in the treatment of BD. Shortly after its efficacy in the treatment of mania was proven, lithium’s efficacy in the prophylaxis of manic, depressive, and mixed episodes of BD has been demonstrated. The use of lithium in the treatment of mania in older patients with BD was supported by a randomized controlled trial (GERI-BD), which compared lithium to divalproex in elderly patients to acute mania. Despite response rates not differ significantly between the lithium and divalproex groups after nine-week, a longitudinal mixed model of improvement favored lithium [113]. More recently, lithium has been found to exert neuroprotective effects, possibly related to its prophylactic action on affective episodes, which may be per se responsible for cognitive impairment in BD patients, but also by means of the inhibition of the glycogen synthase kinase-3 beta, whose overactivation is associated to AD pathophysiology [114]. Importantly, in a recent prospective study, lithium treatment has been associated to a lower incidence of AD and a slowing of cognitive impairment progression [115]. A sample of 61 elderly patients with a diagnosis of amnestic MCI without psychiatric comorbidity was randomized to receive low doses of lithium (0.25-0.50 mmol/L) or a placebo. While participants in the placebo group displayed cognitive and functional decline, patients receiving lithium were cognitively and functionally stable over 24 months and showed greater abilities on memory and attention tests at follow-up. In addition, patients in the treatment group had a 30% increase in cerebrospinal fluid concentrations of the amyloid-beta peptide after 36 months of treatment, indicating less intracerebral storage of amyloid-beta peptide [115]. Intriguingly, a recent epidemiological register-based study found that increases in age-adjusted AD mortality between 2000-2006 and 2009-2015 were negatively correlated with trace lithium levels in public water of Texas Counties, supporting a positive effect of lithium on slowing the AD process [116].

Unfortunately, despite these findings, a dramatic decrease in lithium’s use has been observed in recent decades, especially in elderly patients. This latter tendency can be ascribed both to the low therapeutic index of lithium and to the increasing diffusion of other “mood stabilizers” considered more manageable. As a consequence, scarce attention has been devoted to lithium efficacy in treating BD or behavioral symptoms in patients with dementia. Based on our systematic search, few reports and one clinical trial described the use of lithium in patients with dementia. In a case series of six patients with AD and bvFTD, behavioral symptoms that failed to respond to treatment with antipsychotics improved with low doses of lithium carbonate (ranging from 300 to 600 mg daily) [101]. In addition, one patient with FTD, who was a C9orf72 mutation carrier, had a resolution of mania with antipsychotic and lithium treatment [111]. Nevertheless, in another case nor antipsychotics nor lithium were effective in treating manic symptoms [89]. In a patient with AD, symptoms of agitation, aggression, and wandering have been shown to decrease with lithium 300 mg daily [117]. In another AD patient lithium has been added to the antipsychotic with remission of mania [15]. Moreover, manic symptoms have been shown to stabilize 5 days after taking lithium in patients with VCI [37]. According to these findings, lithium was well-tolerated at serum levels below 0.80 mmol/L. Just one patient had side effects such as sedation and tremor that appeared when the lithium dose exceeded 600 mg daily [101].

More recently, the efficacy of lithium to treat behavioral symptoms in AD was assessed in the 12-week double-blind clinical trial Lit-AD. In this study, low-dose lithium did not differ from placebo in treating agitation, but significantly reduced NPI delusion and irritability/lability. Moreover, lithium was associated to global improvement and reduction of manic symptoms in patients with high Young Mania Rating Scale scores. Importantly, lithium did not differ significantly from placebo on safety outcomes and was not associated to cognitive decline [118].

The tolerability of lithium levels below 0.80 mmol/l was investigated in an open-label study on patients with MCI [119]. After a 1-year treatment, lithium-treated patients reported a rate of side effects similar to those treated with a placebo. The authors observed that side effects were mild and transient and a dose reduction was sufficient to relieve complaints, leading to the conclusion that lithium treatment was safe and well-tolerated at serum concentrations of 0.25-0.5 mmol/L [119]. However, patients with comorbid neurological alterations such as dementia, pre-existing episodes of confusion, EEG abnormalities, and extrapyramidal symptoms (EPS) can be prone to develop frequent and severe lithium-induced neurotoxicity [120-123]. Subjects with neurodegenerative diseases could be vulnerable to neurological side effects such as confusion, disorientation, memory loss, ataxia, and akathisia that may occur even at lithium serum levels within the reference range established for adult patients. Neurotoxicity can be favored by intracellular levels higher than those expected at a given lithium serum concentration in geriatric patients, due to lithium’s pharmacokinetics alterations emerging with age [123, 124]. Importantly, the risk to develop neurotoxicity is particularly increased when lithium is prescribed in combination with antipsychotics [125-127]. When neurotoxicity occurs, symptoms are usually reversible after lithium discontinuation [128], and the reintroduction of lithium at a lower dose is not precluded [129]. However, time for recovery may be prolonged in the elderly and patients with neurological illnesses [129].

### 4.2 Anticonvulsants

Valproate was first approved as an anticonvulsant compound and given its antimanic properties has further achieved the indication for the treatment of BD [130]. Indeed, valproate offers a more manageable alternative to lithium in the treatment of patients with secondary mania related to neurological illnesses [131], showing similar efficacy in acute mania [113]. Moreover, the use of valproate could help to reduce the dose of antipsychotics. Low doses and a slow titration are generally well tolerated. Moreover, valproate confers a lower risk of of drug-drug interaction if compared to other antiepileptics such as carbamazepine. Side effects observed in patients with dementia during treatment with valproate include sedation, gait disturbances, gastrointestinal, urinary tract infections, thrombocytopenia, and tremor [132]. According to a small trial conducted in patients with dementia, valproate may be started at a low dosage (250 mg/day) and slowly titrated (up to 1000 mg/day) [133], bearing in mind that the serum concentrations could not correspond to the therapeutic effect [134]. However, an evaluation of the cost-benefit ratio is necessary before starting a treatment with valproate preparations, since its use in people with AD has been associated to a higher risk of death from causes of dementia [135]. In contrast with what has been shown for lithium [115], there is no evidence that valproate can slow the cognitive or functional progression of AD [136]. Conversely, the use of valproate in bipolar patients has been associated to an increased risk of subsequent dementia [137]. Thus, valproate should be considered to manage mania if lithium is contraindicated or cannot be administered, for example when intravenous therapy is needed. Indeed, most guidelines do not recommend the use of valproate to manage mania and agitation in patients with dementia, unless it is indicated for another condition [138]. However, valproate has been widely prescribed off-label for this purpose based on case reports.

In reviewed studies, mania improved with valproate preparations combined with antipsychotics in two patients with bvFTD [86, 93], one of which also showed cognitive improvements [86], and in one patient with PPA [75]. Valproate preparations successfully treated manic symptoms in two patients with VaD due to CADASIL [54, 68]. Moreover, in a case series of 20 patients with AD, valproate has been found effective and safe for the treatment of behavioral symptoms: 17 out of 20 patients improved with low and flexible doses of valproate, although four required augmentation with other psychotropic medications [139]. In an open-label study, 15 patients with dementia have been treated with valproate preparations, alone or in combination with a second-generation antipsychotic, showing an improvement in physical aggression and irritability [140]. Nevertheless, a meta‐analysis of five studies concluded that valproate preparations had little or no effect on agitated and aggressive behaviors in patients with dementia and higher adverse effects compared to placebo [132]. Importantly, in a small study, which evaluated the efficacy of valproate on mood, psychotic, and behavioral symptoms in old age, a premorbid diagnosis of BD predicted a better response to valproate in the subsample of patients with dementia [141].

The effect of carbamazepine on mania has been also observed in patients with dementia. A case report [142] and two open prospective studies [143, 144] reported improvement of manic-like symptoms with carbamazepine alone or in combination with an antipsychotic, also in patients who had failed to respond to neuroleptic medications. Moreover, a meta-analysis of two RCTs showed significant improvement in behavioral symptoms with carbamazepine monotherapy (300-400 mg/day per over 6 weeks) in comparison to placebo [145]. Both RCTs reported good tolerability of carbamazepine in the short term, whereas one study by Chambers *et al*. [146] observed a worsening in cognitive function and no efficacy on dementia overactivity. The most common neurological side effects are dizziness, sedation, vertigo, ataxia, diplopia, nystagmus, blurred vision, and cognitive impairment [147]. Other relevant side effects include gastrointestinal disturbances, hyponatremia, skin rashes, and blood dyscrasias [148]. Importantly, adverse cognitive effects seem more marked with carbamazepine than with newer antiepileptic drugs [149]. Due to adverse effects and drug interactions involving the P450 cytochrome, the use of carbamazepine is strongly limited in older patients.

Among other anticonvulsants, gabapentin has shown no antimanic properties in young adults, but its favorable toxicity profile along with anxiolytic and antalgic properties make it a suitable medication for elderly patients [150]. Indeed, gabapentin (600-1200 mg/day) combined with antipsychotics resulted well-tolerated and effective in the treatment of mania and potentially helpful in reducing the dose of antipsychotic drugs [150]. A recent systematic review concluded that gabapentin and pregabalin can have a benefit on aggression and agitation in AD and mixed dementia, whereas evidence in FTD is lacking [151]. Notably, gabapentinoids have been associated to a lower risk of death in comparison to valproate and carbamazepine in patients with AD [135]. For this reason, gabapentin and pregabalin could be considered alternative treatments for manic symptoms in AD when antipsychotics and other antiepileptics’ present risks of toxicity and interactions or when comorbid conditions such as anxiety disorders and pain are present.

Finally, lamotrigine is an effective and well-tolerated anticonvulsant for the prevention of mood episodes, particularly depression, which could be used in older adults with BD [152]. In a retrospective medical records review, five patients diagnosed with dementia showed an improvement in manic symptoms and agitation after five months of treatment with lamotrigine (ranging from 100 to 300 mg/day) without side effects and cognitive worsening [153]. Lamotrigine has little influence on the pharmacokinetics of other agents and a generally favorable safety profile [154].

### 4.3 Antipsychotics

Antipsychotics are not approved by the Food and Drug Administration for the treatment of behavioral and psychological symptoms of dementia in the US. As for other countries, among second-generation antipsychotics (SGA) only risperidone received approval for symptomatic management of aggression and severe agitation in the late stages of Alzheimer-type dementia. Nevertheless, dementia represents one of the main reasons for the off-label prescription of antipsychotics. Across different national settings, at least one-fifth of patients affected by dementia is prescribed antipsychotics [155-157]. Dementia is a common diagnosis in patients treated with antipsychotics in primary care in the UK, being diagnosed in approximately 15% of patients receiving antipsychotics overall [158]. In the US, dementia is diagnosed in 25-50% of patients receiving antipsychotics [159]. Organic brain syndromes, including AD, are among the strongest predictors of off-label antipsychotic use, second only to unspecified psychoses [157]. Second-generation antipsychotics (SGAs) are usually preferred, primarily quetiapine, olanzapine, and risperidone [157, 160-164]. Clozapine may be useful for treatment-refractory aggressive behaviors of hospitalized patients with dementia, which have failed to improve with other antipsychotics. Low doses of clozapine (12.5-200 mg/day) seem to rapidly reduce aggressive symptoms favouring early discharge of patients [165]. Prescribed doses of antipsychotics are mostly below the recommended ones for schizophrenia [157, 163], with higher doses being more frequently discontinued [162].

Among patients with dementia, those with behavioural disorders, such as agitation or aggression, and psychotic symptoms are more likely to receive antipsychotics [164, 166-168]. Importantly, BD comorbidity is one of the strongest predictors of antipsychotic medication use in patients with dementia [164, 166, 167]. Nevertheless, no randomized controlled trials of antipsychotic treatments for manic episodes in patients with dementia have been conducted so far. Only a few open-label studies have been conducted in patients with geriatric BD, with some low-quality evidence of efficacy for clozapine [169], risperidone [170], quetiapine [171], and asenapine [172, 173]. Unfortunately, dementia is often included among exclusion criteria in trials of treatments for older adults with BD, while BD patients are almost invariably excluded from trials on dementia-related behavioral disorders.

In randomized, placebo-controlled double-blind trials with patients affected by behavioral and psychological symptoms of dementia, both first-generation antipsychotics (FGAs) and SGAs showed modest efficacy [174, 175], especially in less severe dementia, outpatients, and psychosis [176]. A recent meta-analysis confirmed small effect sizes of aripiprazole, risperidone, and quetiapine, but found no significant differences between olanzapine and placebo on any effectiveness outcomes [177]. In addition, in the largest randomized controlled trial conducted to date in patients with AD-related psychosis, aggression or agitation, atypical antipsychotics were found to improve anger, aggression, and paranoid ideas, but failed to significantly affect cognition, care needs, and quality of life [178]. Conversely, olanzapine was found to worsen overall functioning compared to placebo [178]. Moreover, while antipsychotics marginally outperformed placebo in terms of clinical benefits, the time to discontinuation of treatment due to intolerance, adverse effects, or death significantly favored placebo, and, in about half the patients, olanzapine, quetiapine, and risperidone were discontinued within 8 weeks [179].

Many adverse outcomes of antipsychotic treatment have been reported in patients with dementia. A higher likelihood of venous thromboembolism, stroke and hip fracture has been observed in elderly with dementia exposed to antipsychotics [180]. The risk of venous thromboembolism has been found especially elevated in new users and users of both FGAs and SGAs [181]. A significantly higher risk of cerebrovascular events in patients with AD treated with atypical antipsychotics has also been observed in a meta-analysis of clinical trials [176]. However, there has been some controversy regarding this risk and a large register-based retrospective cohort study of patients diagnosed with AD, VaD or mixed dementia failed to observe an increase in cerebrovascular events in those treated with antipsychotics [182]. Antipsychotic use, instead, has been associated to an increased risk of acute cardiac events [180], head injuries and traumatic brain injuries in patients with AD [183]. In addition, compared to younger patients, the elderly shows a higher risk of long-term antipsychotic-related side effects, such as tardive dyskinesia with FGAs [184, 185] or metabolic syndrome with SGAs [186].

Importantly, in 2005, the US Food and Drug Administration announced that the treatment with SGAs is associated to increased mortality in elderly patients with dementia [187]. The warning, which has been subsequently extended to FGAs [188] based on retrospective studies [189, 190], was initially based on data from seventeen placebo-controlled trials performed with olanzapine, aripiprazole, risperidone, or quetiapine, which showed a nearly 2-fold increase in mortality in the drug-treated group compared to placebo-treated patients. The deaths were mostly caused by heart-related events, such as heart failure and sudden death, or infections, mostly pneumonia [187]. No significant differences in death risk increase were found among atypical antipsychotics in a recent meta-analysis [177], however, quetiapine showed the lowest relative risk in a large retrospective cohort study [191]. Interestingly, the risk of all-cause mortality in older patients with mood disorders initiating antipsychotic treatment has been found to be similar to that of elderly with dementia, which means that increased mortality could be independent of diagnosis and solely related to antipsychotic treatment in older ages [192].

Despite these risks, about two-thirds of antipsychotic-treated old-aged patients with dementia receive long-term treatment in the US [159, 161]. Variations in the prevalence of use of antipsychotic medications can be observed from community to institutional settings, which show higher rates of treatment [166]. Consequently, in 2012, the US Centers for Medicare and Medicaid Services has undertaken a national partnership to improve dementia care, aimed at reducing antipsychotic prescribing in long-term care. The use of antipsychotic treatments in long-stay residents of nursing facilities in the US has indeed declined between 2011 and 2014, especially in patients with dementia. However, antipsychotic use has not decreased in patients with mood disorders [155] and antipsychotics were prescribed in almost two-thirds of subjects with both BD and dementia in nursing homes, even when BD was diagnosed after dementia [155].

Different patterns of prescription were observed in Germany, with a higher number of treatment episodes and a shorter duration in dementia patients suggesting frequent “as-needed” treatment [193]. Importantly, the discontinuation of antipsychotic treatments does not seem to worsen neuropsychiatric symptoms [194] and is feasible in up to 80% of patients treated [195].

In our opinion, antipsychotic treatment benefits should always be balanced with risks and maintenance therapy should be avoided, whenever possible, in patients with dementia-related behavioral symptoms. Low-potency atypical agents could be preferred in patients at high risk of extrapyramidal symptoms. However, as previously suggested [196], low doses of high-potency antipsychotics could be used for short-term treatment of severe manic episodes, with adequate monitoring of side effects.

Since available antipsychotic treatments displayed a limited efficacy and acceptability in dementia-related behaviors, recent studies have been focused on novel biological targets [197, 198]. Promising preliminary results come from a phase III clinical trial on antipsychotics targeting serotonin 5-HT2A receptors: pimavanserin, a selective inverse agonist of the 5-HT2A receptor already approved for treating hallucinations and delusions in patients with Parkinson’s disease, is now under study for AD-related psychosis [197].

### 4.4 Electroconvulsive Therapy

The effectiveness and the safety of electroconvulsive therapy (ECT) have been repeatedly documented in the treatment of mood and psychotic disorders in the geriatric population, where higher response rates have been sometimes found [199]. Many cases of elderly patients with dementia receiving ECT for major depression have been described. Similar response rates were repeatedly observed in depressed patients with and without dementia or MCI both in retrospective [200] and prospective studies [201, 202]. While only a few bipolar depressed patients were included in some of these samples, effective ECT treatment of bipolar depression with comorbid dementia has been also reported in two patients, one affected by FTD [203] and the other by multiple system atrophy [204], respectively showing catatonic and melancholic features. Two patients effectively treated with ECT for agitated delusional depression in the context of AD have also been described [205]. Both patients had neither psychiatric history nor family history of psychiatric disorders [205]. However, their symptoms could be subsumed, in a traditional framework, under the realm of involutional melancholia, which has been equated, by some authors, to bipolar mixed states [206]. ECT has been found effective also in treating mania or agitated behavior in four BD patients affected by advanced dementia [207-209]. Both unilateral [209] and bilateral [207] ECT have been used. Significant improvements in manic symptoms were observed, usually after short courses of ECT, in some cases followed by maintenance courses [209]. In addition, numerous studies suggested ECT effectiveness in the treatment of behavioral excitatory symptoms of dementia, such as aggression and agitation. A systematic review of 17 studies pooled data on 122 patients treated with ECT for dementia-related agitation or aggression [210]. The most common diagnoses of dementia were AD and vascular or mixed dementia. Agitation, screaming, yelling, and physical aggression were the most commonly reported behaviors. In most cases, a bilateral electrode position was used (97 of 122, 80%). Substantial clinical improvements were noted in 88% of cases (107 of 122), often early in ECT course [210]. Interestingly, in more than half of responders who were followed-up after treatment, maintenance ECT was applied (51 of 82, 62%). In the other responders, symptom-free intervals lasted between 3 and 15 months after ECT [210]. In a recent retrospective chart review of 60 patients with dementia presenting with symptoms of aggression or agitation, a significant reduction in agitation after ECT was confirmed, with significant improvements also being reported after 3 and 6 treatments [211]. In addition, a reduction in psychotropic polypharmacy was reported and global functioning significantly improved [211]. Since most studies do not report in detail on mood disorders prior to ECT, it cannot be excluded that the efficacy of ECT in patients with dementia is partly related to mood-stabilizing effects [210].

Common side effects reported are mostly mild and transient, including mild postictal confusion or headache [210]. Nevertheless, postictal agitation after the first treatment has been observed in up to 10% of patients [211]. Delirium has been previously reported in approximately half of depressed patients with dementia treated with ECT [200,201]. However, more recent estimates on agitated patients with dementia do not differ from those usually observed in patients without dementia: according to Verwijk and colleagues [210] delirium was reported in 6 out of 122 patients. Moreover, only one patient out of 60 developed transient delirium in the previously cited retrospective chart review [211]. In this latter study, most patients were treated using right unilateral electrode placement and ultra-brief pulse [211], which has been previously associated to reduced cognitive side effects [212]. Despite these risks, discontinuation of ECT due to adverse events is rarely reported [210, 211].

As for general cognition, improvements in Mini-Mental State Examination (MMSE) scores after ECT have been observed in depressed patients with dementia [200]. However, mixed findings on changes in MMSE scores have been reported in prospective naturalistic studies on depressed patients with cognitive impairment or dementia [201, 202]. Interestingly, the use of AChEIs in patients with dementia undergoing ECT has been proposed to prevent cognitive side effects [202, 213, 214] and two small randomized controlled trials have been conducted on patients without dementia [215, 216]. The continuation of AChEIs given for mild-to-moderate AD during ECT is thus supported and may be protective against ECT-related cognitive side effects. In conclusion, based on the available evidence, the use of ECT could be a valid option for the treatment of dementia-related behavioral symptoms, such as treatment-refractory agitation and aggression, and affective syndromes, including manic, mixed, and depressive states. When treating refractory and potentially life-threatening behaviors, such as self-harm or refusal of food, and liquids, the risk of adverse cognitive effects should be balanced with the potential benefits associated to the treatment. Indeed, while adverse events are generally mild and transient, the therapeutic effects of ECT are longer lasting.

## CONCLUSION

The number of people with dementia is expected to triple to 152 million by 2050 [217]. For this reason and since the majority of these patients usually experience one or more psychiatric disorders over the course of the disease [218], the diagnosis and treatment of mood disorders in the setting of dementia has become of great current importance. BD may be in many cases a prominent clinical manifestation of dementia. Indeed, in elderly patients, a bipolar diathesis can be unmasked by neurodegenerative conditions such as AD, FTD, and VaD. As some authors previously suggested, manic and mixed-labile mood symptoms in the context of cognitive dysfunction, worsening in response to antidepressants, and AChEIs, and showing favorable response to lithium, other mood stabilizers and/or SGAs, may be considered a clinical variant of late-onset bipolarity, especially when associated to premorbid hyperthymic or cyclothymic temperament and a family history of BD [1]. In these cases, the nature of the underlying neurodegenerative conditions should be taken into account to improve treatment strategies and decisions.

In summary, patients with different types of dementia are likely to show different features of mania. The lowest proportion of manic episodes has been documented in patients with AD. However, manic symptoms, such as disinhibition and euphoria, are common, especially in late-stage disease. On the other hand, manic and mixed episodes are more frequently observed in patients VaD. Even a few vascular lesions in subcortical brain regions due to small vessel disease seem sufficient to induce mood instability. Importantly, AChEIs-induced manic episodes have been reported both in patients with AD and vascular alterations. Meanwhile, late-onset bipolarity may represent a prodrome of FTD, especially in its behavioral variant. Indeed, bvFTD may mimic BD in various aspects and the differential diagnosis can be challenging, especially when cognitive signs are absent or subclinical. Moreover, neuroimaging and genetic tests do not always allow the diagnosis at an early stage and a longitudinal follow-up is needed. Nevertheless, differential diagnosis can orient clinicians to effective treatment and overall better management. Notably, irritability and agitation and other abnormal behaviors can significantly improve in FTD patients with medications that target serotoninergic transmission [103,104].

In a personalized medicine perspective, this is an area of great interest for clinical practice that deserves closer attention and further research. Since mania and associated behaviors increased the risk of a negative outcome in patients with dementia [219], it is important to avoid therapeutic nihilism. In order to treat mood disorders in patients with dementia, it seems necessary to provide a carefully planned approach. Psychiatric history, temperament, type, and stage of dementia are variables to consider in choosing the most effective treatment, while carefully evaluating safety and tolerability. In these patients, monotherapy with antipsychotics and antidepressants seems to have limited efficacy, while mood stabilizers and ECT may be more effective and safe [37, 54, 210]. Clinical reports supported the utility of lithium and other mood stabilizers in controlling mixed-manic symptomatology in patients with dementia [101]. In addition to its therapeutic effect on mood alterations, lithium may also exert a disease-modifying effect on the neurobiology of AD [119].

## Figures and Tables

**Table 1:** Manic-like symptoms in patients with Alzheimer’s disease (AD).

**References**	**Study ** **Design**	**N**	**Sample and ** **Setting**	**Age**	**MMSE**	**NPI Factors**	**Prevalence of Manic Symptoms**	**Additional Information**
Frisoni *et al*. (1999) [19]	Cross-sectional	162	Outpatients without a history of psychiatric disorders prior to the onset of dementia	76.4	13.3	Mood, Psychotic, Frontal (euphoria and disinhibition)	Euphoria: 26%; disinhibition: 20.4%	Frontal syndrome prevalence has been shown to be associated to higher education, longer disease duration, and a slower rate of progression
Fuh *et al*. (2001) [20]	Cross-sectional	95	AD outpatient clinic	73.9	12.7	Mood, Psychosis, Psychomotor regulation (agitation, euphoria, irritability), Social engagement	Euphoria: 5%; disinhibition: 34%	Euphoria had an inverse relationship with age. Disinhibition, loaded on Social engagement and had a positive relationship with the severity of dementia
Benoit *et al*. (2003) [21]	Longitudinal (one-year follow-up)	244	AD outpatients	77.2	23.4	MMSE 21-30: Productive, Mood, Sensorial	Euphoria: 4.5%; disinhibition: 10.2%	Frontal syndrome prevalence increased with increasing severity of dementia
		255		77.6	16.7	MMSE 11-20: Mood, Frontal (euphoria and disinhibition), Motor/sensorial	Euphoria: 9.8%; disinhibition: 13.3%	
Aalten *et al*. (2003) [22]	Cross-sectional	199	AD outpatients (excluded if living in a nursing home)	76.4	18.2	Mood/apathy, Psychosis, Hyperactivity (agitation, euphoria, irritability, disinhibition, aberrant motor behavior)	Euphoria: 7%; disinhibition: 12.6%	-
Mirakhur *et al*. (2004) [16]	Retrospective (caregiver interview)	435	Outpatient memory-clinic	78	13	Affective, Physical behaviour, Psychosis, Hypomania (euphoria and disinhibition)	Euphoria: 16.6%; disinhibition: 29.5%	Agitation/aggression/irritability clustered with depressive symptoms
Hollingworth *et al*. (2006) [23]	Cross-sectional	1120	AD late-onset volunteers	81.2	12.8	Behavioral dyscontrol (euphoria, disinhibition, aberrant motor behavior, sleep/appetite disturbances), Psychosis, Mood, Agitation	Euphoria: 10.5%; disinhibition: 31.4%	A higher score in behavioral dyscontrol was associated to female sex, younger age at the onset, and more severe cognitive impairment.
Spalletta *et al*. (2010) [18]	Cross-sectional, multicenter	1015	Untreated AD outpatients	73.3	18.3	Apathy, Affective, Psychomotor, Manic (disinhibition and euphoria), Psychotic	Euphoria and/or disinhibition: 7.7%	Increased occurrence/severity of manic syndrome with cognitive decline
Palmer *et al*. (2011) [17]	Longitudinal (one-year median follow-up)	177	Memory-clinic AD outpatients	73.1	19.4	Apathy, Affective, Psychomotor, Manic (disinhibition and euphoria), Psychotic	Euphoria and/or disinhibition: 7.9%	The manic syndrome was associated with an increased risk of cognitive decline.

**Abbreviations:** AD = Alzheimer’s disease, MMSE = Mini-mental state examination, NPI = Neuropsychiatric inventory.

**Table 2:** Patients with Alzheimer’s disease (AD) reporting mania or hypomania after acetylcholinesterase inhibitor treatment.

**References**	**Age**	**Sex**	**Diagnosis**	**MD (Onset)**	**AChEI**	**DD**	**Concomitant Drugs (Duration)**	**Time to Mania**	**PS**	**Time to Resolution**	**Treatment**
Ehrt *et al*. (2011) [27]	81	F	AD	BD 1 (40 y)	Rivastigmine	4.6 mg	Venlafaxine, lithium (2 y)	1 week	No	1 week	None
Ehrt *et al*. (2011) [27]	76	M	AD	BD 1 (adolescence)	Galantamine	16 mg	Escitalopram, lithium (1 y)	9 days after increase	No	2 weeks	None
Tseng and Tzeng (2012) [28]	79	M	AD	None	Rivastigmine	3 mg	None	3 days	No	2 days	None
Hategan and Bourgeois (2016) [30]	72	F	Mixed dementia	MDE (64 y)	Donepezil	10 mg	Duloxetine (8 y)	1 week after increase	Yes	17 days	Risperidone
Jalal *et al*. (2017) [29]	79	F	Mixed dementia	BD 1 (74 y)	Galantamine	8 mg	Divalproex	2 weeks	No	4 weeks	Divalproex increase and risperidone
Faisal *et al*. (2017) [31]	92	F	AD	Depressive symptoms	Donepezil	10 mg	Memantine	Many months	Yes	Rapid	Quetiapine (failed trial)

**Abbreviations:** AChEI = Acetylcholinesterase inhibitor, AD = Alzheimer’s disease, BD 1 = Bipolar disorder type 1, DD = Daily dose, F = Female; M = male, MDE: Major depressive episode, MD = Mood disorder, PS = Psychotic symptoms, y = years.

**Table 3:** Mania in patients with Vascular Dementia (VaD) and Vascular Cognitive Impairment (VCI).

**References**	**Age**	**Sex**	**Family History**	**Somatic Comorbidity**	**Antecedents (onset)**	**CVE**	**VCI (onset)**	**CT/MRI Findings**	**Concomitant Drugs**	**Treatments**
**Cortico-subcortical VCI (Due to Large Vessel Disease)**										
Damodaran *et al*. (1994) [55]	40	M	None	-	Depressive episode (38 y)	-	Gradual cognitive decline (40 y)	Two small lesions in the frontal lobes, one in the deep left parietal lobe	Imipramine, thioridazine	Antipsychotics and lithium (wandering and confusion appeared)
Watanabe *et al*. (2006) [52]	64	M	**-**	Hypertension, diabetes, cardiac diseases	BD 2 (40 y)	Left frontotemporal lobe infarction (62 y)	VaD (2 months before mania)	Left frontotemporal lobe encephalomalacia; moderate cortical atrophy	**-**	**-**
Duggal and Singh (2009) [57]	47	M	None	Diabetes	Chronic depression (35 y)	Left inferior frontal region infarct (46 y)	VaD (5 months before mania)	Encephalomalacia and ischemic changes in WM and basal ganglia	Benzodiazepines, opioids, bupropion (chronic treatment)	Ziprasidone, carbamazepine, donepezil, discontinuation of bupropion
Duan *et al*. (2018) [37]	73	F	None	Atrial fibrillation	Recurrent depression (33 y)	Right cerebellar hemisphere, left occipital lobe, and left pons cerebral infarctions (69 y)	Vascular cognitive impairment (73 y)	Multiple encephalomalacias; ischemic changes in bilateral periventricular regions and bilateral centrum semiovale	Venlafaxine and quetiapine (chronic treatment)	Discontinuation of venlafaxine; quetiapine increase (no response); donepezil and memantine (worsening); resolution with lithium carbonate
**Subcortical VCI (Due to Small Vessel Disease)**										
Iijima *et al*. (1993) [51]	65	F	Mental retardation	-	None	TIAs (> 55 y)	VaD (69 y)	Bilateral and symmetrical WM ischemic changes (Binswanger’s disease)	**-**	-
Senturk *et al*. (2006) [56]	60	F	Major depression	-	Major depression (59 y)	-	Cognitive dysfunction (60 y); VaD (61 y)	Atrophy and multiple ischemic gliotic lesions in the periventricular subcortical WM, basal ganglia, and pons	Antidepressant discontinuation 3 months before mania	VPA and BDZ (partial response); resolution with clozapine (depressive symptoms appeared)
**VCI due to CADASIL**										
Kumar and Mahr (1997) [58]	55	M	CADASIL	-	Alcohol abuse	-	Little evidence of cognitive impairment	Ischemic changes in the periventricular and subcortical WM of both cerebral hemispheres, corona radiata, centrum semiovale bilaterally, both basal ganglia, pons, and thalami	-	-
Leyhe *et al*. (2005) [59]	60	M	Stroke, dementia, migraine	-	-	-	Executive and attention deficits (60 y)	Severe confluent leukoencephalopathy with single lacunar infarcts and microbleedings in the basal ganglia	-	Pipamperone
Park *et al*. (2014) [53]	53	F	Depression, suicide	Hypertension, diabetes	Personality changes started (48 y)	-	Executive and attention deficits	Severe leukoencephalopathy, ischemic changes in the peri- ventricular white matter, basal ganglia, thalamus, and the external capsule	Antidepressants and hypnotics (chronic treatment)	Quetiapine and BDZ (partial response)
Bangash and Saad (2016) [54]	58	F	None	-	None	TIAs (57 y)	Memory and visuospatial reasoning deficits (58 y); VaD (59 y)	Ischemic changes in the subcortical, periventricular and deep WM, in the pons, bilateral inferior temporal lobe, and posterior fossa	None	Olanzapine (relapse); aripiprazole (relapse); resolution with VPA
Uppal *et al*. (2020) [60]	64	F	-	TBI with subdural hematomas	Depression, alcoholism and personality change (> 40 y)	-	Executive deficits	Ischemic changes in the supratentorial and infratentorial WM, chronic haemorrhagic infarctions in the frontal, and temporal lobe	Amphetamine dextroamphetamine (chronic treatment)	Discontinuation of stimulants; quetiapine, and lithium
Okamoto *et al*. (2021) [68]	80	F	-	Hypertension, uterine fibroids	Personality changes (79 y)	-	Attention deficits and poor judgment (79 y)	Chronic ischemic changes in bilateral temporal and occipital lobes, right subcortical frontal lobes, and right illuminating hemisphere. Atrophy of the medial temporal lobe	-	Resolution with VPA

**Abbreviations:** BD 2 = Bipolar disorder type 2, BDZ = Benzodiazepines, CADASIL = Cerebral autosomal dominant arteriopathy with subcortical infarcts and leukoencephalopathy, CT = Computerized tomography, CVE = Cerebrovascular event, F = Female; M = Male, MRI = Magnetic resonance imaging, TBI = Traumatic brain injury, TIA = Transient ischemic attack, VaD = Vascular dementia, VCI = Vascular cognitive impairment, VPA = Valproic acid, WM = White matter, y = years.

**Table 4:** Patients with bipolar disorder (BD) and subsequent or concomitant frontotemporal dementia (FTD).

**References**	**Sex**	**Psychiatric Antecedents**	**Age at BD**	**Age at FTD**	**FTD**	**Symptoms**	**Neuroimaging**	**MMSE**	**Neuropsychological Tests**	**CSF ** **Analysis**	**Genetic Tests**
**Young Adulthood BD (< 40 years) Followed by FTD**											
Borges *et al*. (2019) [78]	F	BD 1	16	78	bvFTD	Progressive impulsivity, verbal, and physical aggression, short-term memory loss, perseverative behaviors, perambulation, persecutory delusions, disorientation, hyporexia, occupational impairment	MRI: brain atrophy with frontotemporal predominance and ischemic microangiopathy SPECT: moderate/severe bilateral frontotemporal hypoperfusion/activation	17	Impaired executive functions, language, memory, and attention	-	-
Cerami *et al*. (2011) [80]	M	BSD (retrospective diagnosis)	-	57	bvFTD	Apathy, lack of motivation, retirement from social/community life, followed by impulsivity, aggressiveness, insomnia, motor hyperactivity, concentration difficulties	MRI: severe cortico-subcortical atrophy, predominantly affecting right frontal and temporoparietal areas	28	Dysexecutive profile	-	GRN mutation
Papazacharias *et al*. (2017) [75]	F	BD 1	20	56	PPA	Early language impairment, poor coordination, aphasia, dysgraphia, disinhibition, weight gain due to the craving for sweet foods, decrease in personal hygiene, stereotyped motor behavior, disturbances in recognizing familiar faces	MRI: white matter gliosis in bilateral subcortical frontal areas, diffuse cortical atrophy in bilateral frontal and temporal areas SPECT: hypoperfusion in bilateral frontal and temporal areas	23	Dysexecutive profile	Normal	Normal
Papazacharias *et al*. (2017) [75]	M	BD 2	28	53	FTD with parkinsonism	Neurological signs (dysarthria, ataxia)	MRI: cortical atrophy in frontal and temporal areas SPECT: hypoperfusion in bilateral frontal and temporal areas	22	Dysexecutive profile	Mild increase of tau proteins	Polymorphism associated to FTD on exon 12 of chromosome 17 (3’UTR+78C/T)
Pavlovic *et al*. (2011) [77]	F	BD 1	33	68	bvFTD	The manic episode followed by low mood, anhedonia, lethargy, then aggressiveness, cognitive impairment, and decline in social/personal conduct, hyperorality, craving for sweets, stereotyped behavior, utilization behavior aspontaneity, intermittent mutism	CT: mild widening of frontal sulci and enlarged frontal horns of lateral ventricles. MRI: technically limited but confirmed similar findings. SPECT: reduced blood flow in both frontal and temporal lobes, more marked on the left side	17 to 24	-	-	-
Poletti *et al*. (2013) [76]	M	BD (hypersexual in manic phases)	Young adulthood	71	bvFTD	Poor hygiene and personal care, disinhibition, prodigality	Not reported	30	Impaired executive functions (decision- making only)	-	-
Velakoulis *et al*. (2009) [79]	M	BD	34	39	FTD	-	-	-	-	-	-
**Late-onset BD (> 40 Years) Followed by FTD**											
Cerami *et al*. (2011) [80]	M	BD 2	42	60	PPA	Dysarthria, reduced fluency, anomias, phonological errors, dropping of function words, and verbal perseverations	MRI: cerebral atrophy, predominantly affecting left frontotemporal and perisylvian areas	-	-	-	GRN mutation
Floris *et al*. (2014) [84]	M	BD 1	42	64	bvFTD	Repeated euphoric manic and hypomanic episodes followed by one episode of sexual disinhibition, delusional fixed ideas, and repetitive behaviors (64 years)	MRI: bilateral frontotemporal atrophy, prominent in frontal areas SPECT: reduction of uptake in the left frontotemporal and right frontoparietal regions	-	Marked deficits in attention, executive function and working memory, anomia, and verbal fluency dysfunction	-	C9ORF72 gene (>70 repeats)
Martins *et al*. (2018) [82]	F	BD 1	75	85	bvFTD	The manic episode with persecutory delusions and auditory hallucinations, followed by mood swings	MRI: diffuse cortical atrophy with frontal predominance; hippocampi were only slightly reduced SPECT: hypoperfusion of the frontal lobes	16	Visuospatial disabilities, dysexecutive profile	Normal	-
Monji *et al*. (2014) [81]	M	BD 1	46-47	52	bvFTD	Depressive symptoms (42 years), followed by hypomanic symptoms including hypersexual talk, public masturbation at 46, and apathy at 47	SPECT: predominant frontal hypoperfusion MRI: predominant frontal brain atrophy	-	Frontal lobe hypofunction (WCST)	-	-
Rubino *et al*. (2017) [83]	M	BD 1	55	70	bvFTD	Less extroversion, indifference, hyperphagia, followed by apathy, retirement from social and leisure activities	CT: asymmetrical brain ventricles and mild frontotemporal atrophy 18-FDG PET: marked hypometabolism in bilateral frontotemporal areas	22	Selective attention, verbal memory, and executive functions deficits	Normal	A c.1639 C>T variant in the exon 12 of the GRN gene
**The First Manic Episode at FTD Onset**											
Bretag-Norris (2019) [89]	F	Depression	-	72	bvFTD	The manic episode preceded by personality and behavioral change (incarcerations oversea for driving and drug offenses, property damage, and loss of money 18 months before)	MRI: moderate-severe frontotemporal parenchymal brain volume loss SPECT: marked bilateral frontotemporal hypoperfusion	-	Dysexecutive profile	-	-
Dionisie *et al*. (2020) [91]	F	None	-	48	bvFTD	Childish behavior, various and repeated verbal and physical conflicts with different people, dromomania, excessive spending, disinhibition	CT: significant global cerebral atrophy MRI: significant bilateral frontotemporal atrophy (the temporal lobes were more severely affected than the frontal lobes)	19	-	-	-
Galvez-Andres *et al*. (2007) [93]	F	None	-	59-62	bvFTD	Progressive personality change, neglect of personal hygiene, hoarding, suspiciousness, wandering, followed by a manic episode, then anhedonia, apathy, anxiety, insomnia, somatic complaints	MRI: normal. 18-FDG PET: normal	24	Dysexecutive profile	-	-
Ibáñez (2012) [90]	M	BD 1	44	45	bvFTD	Manic episode with psychotic features	MRI: progressive atrophy in temporoparietal regions 18-FDG PET-CT: diffuse hypometabolism, with strikingly decreased metabolic activity symmetrically in bilateral frontal and anterior temporal lobes	-	-	-	-
Kerstein *et al*. (2013) [87]	M	Subsyndromal hypomania (retrospective diagnosis)	-	65	bvFTD	Apathy, anhedonia, and lack of energy followed after 3 years by a manic episode with disinhibited sexual behavior, lability	Unremarkable	20	Weakness in visuospatial abilities and impairments in mental processing speed, working memory, executive functions	-	-
Payman *et al*. (2019) [92]	M	None	-	67	bvFTD	Mania with psychotic features preceded by professional misconduct (misappropriating money, falsifying documents, lying to investigators) 18 months before, and followed by apathy, verbal and manual stereotypies	MRI: diffuse cerebral tissue loss predominantly in the frontotemporal lobes 18-FDG PET: hypometabolism in the anterolateral frontal lobes and anterior cingulate gyrus	22	Prominent and severe executive dysfunction and impaired new learning	-	C9ORF72 mutation
Prevezanos *et al*. (2017) [85]	M	None	-	76	bvFTD	Loosening of associations and ample profanity, night-wandering, followed by diminished volition, and increased reliance on caregivers for planning activities of daily living	CT: significant atrophy involving the frontal and temporal lobes	30	Visuospatial disabilities, dysexecutive profile	-	-
Saridin *et al*. (2019) [88]	M	None	-	69	FTD-ALS	Manic episode (meddling and fight picking with spouse and authorities; disinhibition, incoherent thought); one year later apathy and reduced empathy, impaired speech, progressive gait instability, and hand weakness	MRI: age-related atrophy and white matter intensities (no changes after two years)	-	Dysexecutive profile	A specific profile with a slightly decreased amyloid-beta concentration, normal levels of t-tau and p-tau	C9ORF72 repeat expansion > 30
Shah (2013) [94]	M	None	54	55	bvFTD	Irritability, behavioural changes (excessive time on the phone, extra measures for grooming, stereotyped interests, incongruous planning, decreased sleep and increased demand for specific food items, hypersexuality, alcohol intake, and smoking)	MRI: diffuse cerebral atrophy, principally in the frontal and temporal area and greater on the right side	21	Significant impairment in remote and recent memory, poor perceptual-motor function (BVMGT), and significant executive dysfunction (WCST)	-	-
Vorspan *et al*. (2012) [86]	F	Two MDEs	-	54	bvFTD	Mania with echolalia, echopraxia, amnesia, hyperorality, followed by apathy, mutism, motor retardation, anosognosia, then euthymia (two additional episodes)	CT: cortical atrophy SPECT: anterior temporal and frontal lobe hypoperfusion. MRI: frontal atrophy	-	Dysexecutive profile with impaired working memory and attention, mild impulsivity, reduced mental flexibility	Normal	-
**Iatrogenic Mania in FTD**											
Thorlacius-Ussing *et al*. (2020) [111]	M	None	-	54-58	bvFTD with parkinsonism	Irritability, lack of empathy, and social withdrawal, followed by mild akinetic-rigid parkinsonism with right-sided bradykinesia and rigidity. Worsening of manic symptoms after levodopa	18-FDG PET: widespread reduced metabolic activity in frontal and parieto-temporal areas with a left-sided predominance DAT-SPECT: significant tracer binding asymmetry with decreased binding especially on the left side (early loss of functional nigrostriatal dopaminergic neuron terminals)	-	Impaired executive functions and emotional recognition	Normal	Heterozygous for hexanucleotide repeat expansion (G4C2) within C9orf72

**Abbreviations:** 18-FDG PET = 18-Fluoro-2-deoxy-d-glucose positron emission tomography, BD = Bipolar disorder, BD 1 = Bipolar disorder type 1, BD 2 = Bipolar disorder type 2, BSD = Bipolar spectrum disorder, BVMGT = Bender visual-motor gestalt test, DAT-SPECT: Dopamine transporter - single photon emission computed tomography, F = female, FAB: Frontal assessment battery, MDEs = Major depressive episodes, MMSE = Mini-mental state examination, MRI = Magnetic resonance imaging, CSF = Cerebrospinal fluid, CT = Computed tomography, M = male, NPI: Neuropsychiatric inventory, SPECT = Single photon emission computed tomography, WCST = Wisconsin card-sorting test.

## LIST OF ABBREVIATIONS

BD 2: Bipolar Disorder Type 2
BDZ: Benzodiazepines
CADASIL: Cerebral Autosomal Dominant Arteriopathy With Subcortical Infarcts and Leukoence-phalopathy
CT: Computerized Tomography
CVE: Cerebrovascular Event
F: Female
M: Male
MRI: Magnetic Resonance Imaging
TBI: Traumatic Brain Injury
TIA: Transient Ischemic Attack
VaD: Vascular Dementia
VCI: Vascular Cognitive Impairment
VPA: Valproic Acid
WM: White Matter
y: Years

## STANDARDS OF REPORTING

PRISMA guidelines were followed in this study.

## SUPPLEMENTARY MATERIAL

PRISMA checklist is available as supplementary material on the publisher’s website along with the published article.
